# Long noncoding RNA SNHG6 mainly functions as a competing endogenous RNA in human tumors

**DOI:** 10.1186/s12935-020-01303-x

**Published:** 2020-06-06

**Authors:** Hui-shan Wang, Wen Zhang, Han-long Zhu, Quan-peng Li, Lin Miao

**Affiliations:** 1 Nanjing Medical University, Nanjing, Jiangsu Province China; 2 Medical Center for Digestive Diseases, The Second Affiliated Hospital of Nanjing Medical University, 121 Jiangjiayuan, Nanjing, 210011 Jiangsu Province China

**Keywords:** Long non-coding RNA, SNHG6, Competing endogenous RNA, Tumors

## Abstract

Increased expression of the small nucleolar RNA host gene 6 (SNHG6) has been reported in different cancers, such as hepatocellular carcinoma, colorectal cancer, and lung cancer. The high expression level of SNHG6 is associated with tumor progression and poor prognosis. This paper provides an overview of recent studies on the oncogenic role and potential clinical utilities of SNHG6. Upregulated SNHG6 arrests tumor cell cycle and reduces apoptosis but promotes migration, invasion, metastasis, epithelial-mesenchymal transition (EMT), and chemoresistance in tumors. Mechanically, SNHG6 primarily sponges tumor suppressor microRNA (miRNA), functioning as a competing endogenous RNA. Once sponged, miRNA is unable to degrade, silence, or hamper the translation of its downstream, mostly oncogenic genes, ultimately driving cancer-related processes. Thus, SNHG6 might serve as a biomarker for cancer diagnosis and prognosis.

## Background

With technical progress in sequencing technologies, an increasing number of noncoding RNAs (ncRNAs) have been discovered in the last several years. Long ncRNA (lncRNA), > 200 nucleotides in length, has been reported to participate in a variety of biological processes, such as regulation of gene expression, subcellular architecture, and stabilization of protein complexes [1]. The role of lncRNA in physiology and pathophysiology was also reported [2]. Mechanistically, lncRNA often sponges many different types of miRNAs, acting as competing endogenous RNA (ceRNA) to realize its function. ceRNAs are transcripts competing for shared microRNA (miRNA) by complementary sequences [3]. MicroRNAs (miRNAs), a class of small ncRNAs typically 22 nucleotides in length, usually bind to the 3′-untranslated region of the target-gene mRNA, and once sponged, miRNA is unable to repress target mRNA translation or induce mRNA degradation [4]. The hypothesis of “ceRNA” was posed officially in 2011 when Salmena et al. expounded that RNA transcripts, similar to mRNAs, transcribed pseudogenes or lncRNAs containing miRNA-response elements (MREs), function as ceRNA and de-repress the activity of other RNAs with similar MREs by competing for the same miRNA in the available miRNA pool [5].

The subject of ncRNA functioning as ceRNA in tumor formation and progression has been extensively explored, and many ncRNAs have been investigated as miRNA sponges in a variety of cancers. Such ncRNAs include GAS5 in pancreatic cancer [6], ZFAS1 in colon adenocarcinoma [7], and MALAT1 in endometrioid endometrial carcinoma [8]. This review will summarize the most recent findings on SNHG6, focusing on the effect of its upregulation and its role as ceRNA in tumor progression.

## SNHG6—A novel player in human tumors

Human SNHG6 (Ensembl: ENSG00000245910) is the housekeeping gene of the 5′TOP family, which can encode two kinds of noncoding RNAs, namely, U87 C/D box snoRNA, synthesized by the second intron, and SNHG6 RNA, encoded by exons. SNHG6 is located in chromosome 8q13.1 and consists of five transcripts (i.e., SNHG6-201 to SNHG6-205). SNHG6 is localized preferentially in the cytoplasm by cytoplasmic and nuclear RNA fractions from some cancer cells, such as hepatocellular cells and colorectal cancer cells [9, 10]. According to recent studies, SNHG6 is overexpressed in cancer tissues compared with the corresponding noncancerous tissues as well as in different cancer cell lines [11–13]. Upregulated SNHG6 relates to advanced tumor progression and short survival in patients [14, 15]. SNHG6 is responsible for cell proliferation, migration, invasion, reduced apoptosis in vitro, increased tumor size, and increased metastases in vivo [16, 17].

The next section will mainly discuss the information gained in recent years about the role of SNHG6 in some relative frequent cancer types, such as hepatocellular carcinoma (HCC), colorectal cancer (CRC), gastric cancer (GC), esophageal squamous cell carcinoma (ESCC), lung adenocarcinoma (LUAD), breast cancer (BC), bladder cancer, glioma, and osteosarcoma.

### Hepatocellular carcinoma (HCC)

SNHG6 is upregulated in HCC tissues and cell lines. Overexpressed SNHG6 is tightly related to tumor development and poor survival [9, 18]. Cao et al. reported that five SNHG6 transcripts differentially expressed in HCC tissues, while only SNHG6-003 exerted an oncogenic function, which serves as ceRNA by binding to miR-26a/b, thereby regulating transforming growth factor-β-activated kinase 1, an oncogene of HCC [18]. Another study demonstrated that SNHG6 plays an oncogenic role in liver tumorigenesis by activating the TGF-β1/SMAD signaling pathway and upregulating zinc finger E-box-binding homeobox1 (ZEB1) via effectively sponging miR-101-3p, resulting in epithelial–mesenchymal transition (EMT) [9]. Chen et al. recently showed that SNHG6 promotes HCC cell proliferation via competitively binding let-7c-5p and thereby regulating the expression of c-Myc [19]. Besides, SNHG6 could also activate SERPINH1 expression by competitive binding to miR-139-5p in HCC, which is verified by Wu et al. [17]. Guo and his colleagues shed light on the role of SNHG6 in genome-wide hypomethylation in hepatocellular cells. They verified that SNHG6, negatively correlated with the steady-state S-adenosylmethionine (SAMe) concentration in vivo and in vitro, suppressed MAT1A protein expression by activating the miR-1297/FUS pathway [20]. Methionine adenosyltransferase (MAT) are essential enzymes that catalyze SAMe formation, and MAT2A is expressed in the proliferating liver during dedifferentiation and in cancer, while MAT1A is expressed in quiescent adult hepatocytes [21, 22]. Interestingly, the effect of SNHG6 on genome-wide methylation was inhibited by exogenous SAMe within a certain concentration range, indicating the potential benefit of SAMe for treatment of liver cancer. In summary, these findings demonstrated that SNHG6 could promote progression of HCC by acting as a ceRNA from different aspects.

### Colorectal cancer (CRC)

In the last 3 years, scholars have explored the role of SNHG6 in CRC. Generally, SNHG6 was found to be upregulated in CRC tissues and cell lines and responsible for high tumor grades and poor patient survival. Highly expressed SNHG6 could enhance CRC cell proliferation, invasion, and migration. SNHG6 could also act as miRNA sponge to induce the dysfunction of the following miRNAs: (a) miR-760 [23], (b) miR-101-3p [10, 12], (c) miR-214-3p [24], (d) miR-26a-5p, miR-26b-5p [24], and (e) miR-181a-5p [24]. Zhu et al. demonstrated the role of miR-760 in CRC. They illustrated that miR-760, as a direct target of SNHG6, could reverse the inhibitory effect of SNHG6 knockdown on CRC progression by targeting forkhead box C1 [23]. The relationship between SNHG6 and miR-101-3p is indentified by two study groups. In Wang’s study, they found that SNHG6 sponges miR-101-3p, inducing an upregulated expression of ZEB1, which is a key transcription factor in EMT. They also proved that SNHG6 could activate the TGF-β/Smad pathway by binding to UPF1 in CRC cells [10]. While Shao’ data showed that SNHG6 could regulate the progression of CRC via modulating the expression levels of miR-101-3p and the activity of Wnt/beta-catenin signaling [12]. Xu et al. proved that SNHG6 could interact with miR-214 and miR-26a/b and regulate their common target-EZH2 [24], while another group also showed the relationship between SNHG6 and miR-26a. In their study, SNHG6 promotes chemoresistance of CRC cells through ULK1-induced autophagy by sponging miR-26a-5p [26]. For miR-181a-5p, Yu et al. demonstrated that E2F5, as a direct target of this miRNA, is upregulated, resulting in increased CRC proliferation by regulating the cell cycle [25]. Moreover, Li et al. proved that SNHG6 could directly suppress p21 expression by recruiting EZH2 to the p21 promoter in CRCs [27]. However, in Meng’ study, they verified that SNHG6 was downregulated in colorectal cancer tissues, suppress ETS1 via the PI3K/AKT/mTOR pathway to inhibit CRC cell proliferation and metastasis [28]. Due to only 30 colon tumors and adjacent non-tumor tissues samples were detected, we think the results is not so convincing compared with many other studies with positive results. From the above, the authors concluded that SNHG6 was acting in an oncogenic role by binding multiple miRNAs and abrogating its tumor-suppressive function in CRC progression.

### Gastric cancer (GC)

Studies have reported some connections between SNHG6 and GC. Similar to its role in HCC, SNHG6 is upregulated in GC and its high expression influences cancer cell characteristics, such as cell growth, migration capacity, and EMT. Yan et al. [29] revealed that SNHG6-promoted cell growth could be due to its influence on cell cycle through interacting with PRC2 and epigenetic silencing p27, whereas SNHG6-accelerated migration could be through miR-101-3p sponging, thereby regulating ZEB1. Li’s study indicated that SNHG6 facilitates GC progression by upregulating p21 through activation of the JNK pathway and suppression of EZH2 [30]. Therefore, these data have revealed that SNHG6 plays an important role in progression of GC through targeting key promoters in the cell cycle such as P21 and P27.

### Esophageal squamous cell carcinoma (ESCC)

Fan’s and Zhang’s groups both found that SNHG6 expression is significantly increased in ESCC tissues and is associated with tumor size and TNM stage [31, 32]. These two groups both found that SNHG6 knockdown can inhibit proliferative, colony‑forming abilities, and induce the apoptosis of ESCC cells. They also concluded that SNHG6 exerts oncogenic function in ESCC and may be a potential diagnostic marker for this cancer. In Du’ study, they demonstrated that SNHG6 promoted the proliferation, migration, and invasion of ESCC cells through regulating miR-186-5p/HIF1α axis [33]. Above all, the results may provide a novel therapeutic target–SNHG6 for ESCC. Besides, more functions and detailed molecular mechanisms of SNHG6 in ESCC need to be explored.

### Lung cancer (LC)

Several studies all found that SNHG6 expression was also significantly increased in non-small cell lung cancer (NSCLC) tissues and cell lines and its high expression was correlated with malignant features of NSCLC. In Geng’ study, knockdown of SNHG6 significantly depressed the proliferation vitality and migration activity of NSCLC cells in vitro. Research on mechanisms illustrated that SNHG6 regulates ETS1 signaling via miR-944 and miR-181d-5p [34]. In Dong’ study, SNHG6 significantly promoted proliferation and inhibited apoptosis of NSCLC cells. Mechanism research demonstrated that SNHG6 regulates miR-490-3p/RSF1 axis [35]. Li and his colleagues found that increased expression of SNHG6 was associated with pathological stage and lymph node infiltration, and acted as an independent prognostic factor of tumor recurrence in patients with NSCLC. Silencing SNHG6 expression repressed cell growth and invasion in vitro and in vivo. Mechanically, SNHG6 was identified to regulate CDYL expression by acting as a sponge of miR-101-3p [36]. More than that, Liang et al. found that SNHG6 expression is higher in lung adenocarcinoma (LAOD) tissues than in adjacent non-tumor tissues, and its overexpression is related to tumor development and poor survival in patients. Functionally and mechanically, SNHG6 promotes cell cycle progression, cell proliferation, migration, invasion, and EMT by acting as ceRNA via competitively binding to miR-26a-5p, thereby activating E2F7 [37]. To summarize, the authors demonstrated that SNHG6 were involved in progression of lung cancer by regulating multiple miRNAs, representing promising targeted therapeutic strategies against NSCLC.

### Breast cancer (BC)

Recent TCGA data analysis showed that SNHG6 might serve as a potential prognostic marker for BC without further experimental validation [38]. Two study groups showed that high SNHG6 expression increases BC cell proliferation by targeting miR-26a-5p, and miR-26a-5p targets respectively to MAPK6 [39] and VASP [40]. Therefore, the authors conclude that SNHG6 participates in BC development through the miR-26a-5p/MAPK6 and miR-26a-5p/VASP pathway. Another group suggested that SNHG6 could also be involved in ionizing radiation‑induced stress response in a tumor protein p53‑dependent manner [41]. From these results, the authors concluded that SNHG6 was served as an oncogene by binding miR-26a in BC progression.

### Bladder cancer

High expression of SNHG6 in bladder cancer cells was discovered by Wang et al. [42]. Their data suggested that overexpressed SNHG6 induces EMT through upregulating Snail1/2 and promotes migration and invasion of bladder cancer cells by sponging miR‐125b, thereby activating the target gene of miR-125b-novel (nua) kinase family 1 (NUAK1), also known as ARK5. Thus, SNHG6 accelerates bladder cancer cell progression through miR‐125b/NUAK1 and miR‐125b/Snail1/2 pathways.

### Glioma

Meng et al. found that SNHG6 also upregulates in glioma tissues and cells compared with normal brain tissues and cells [43]. In their research, SNHG6 promotes glioma cell proliferation, migration, and EMT and reduces apoptosis by downregulating miR-101-3p. Anti-miR-101-3p and miR-101-3p mimic rescue the effects of si-SNHG6 on cell malignancy. However, details about the target gene of miR-101-3p are not shown in this study. Another study carried out by Cai and his team workers detected that upregulated SNHG6 is responsible for glioma cell proliferation, which is consistent with Meng’s results [44], while silencing SNHG6 can induce cell cycle arrest by upregulating p21.

### Osteosarcoma

Ruan et al. revealed that upregulated SNHG6 predicts poor survival and advanced TNM stage for patients with osteosarcoma [45]. They investigated that si-SNHG6 represses cell proliferation by arresting cell cycle in the G0/G1 phase and inducing cell apoptosis. Mechanically, SNHG6 is negatively correlated with p21 and Kruppel Like Factor 2 (KLF2), which is a target gene interacting with ncRNAs in malignancies [46]. p21 and KLF2 play vital roles in osteosarcoma progression, such as miR-95-3p/p21 axis [47], and involvement of KLF2 in drug resistance to doxorubicin [48]. In Zhu’s study, they found that SNHG6 could competitively sponging miR-26a-5p thereby regulating ULK1, and induced cell apoptosis and autophagy by targeting caspase3 and ATF3 [49]. Thus, SNHG6 is involved in osteosarcoma progression and may serve as an oncogene.

## Conclusion and future perspectives

**Fig. 1:**
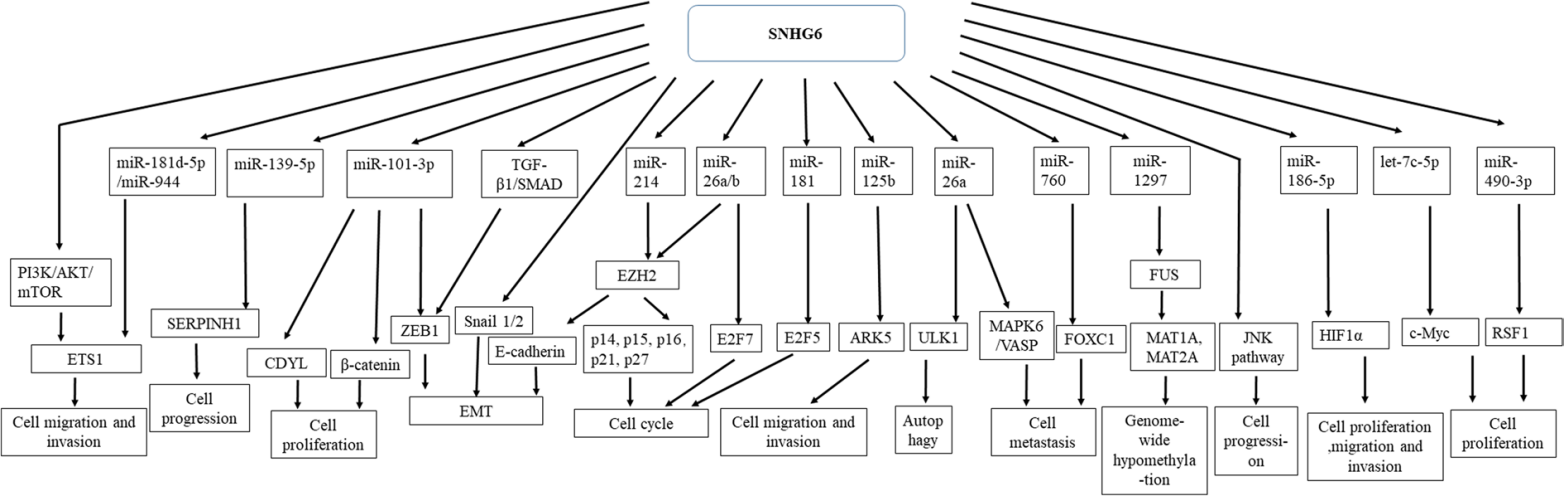
The role and underlying molecular mechanisms of SNHG6 in multiple human cancers. SNHG6 could get involved in the progress of cell migration and invasion, cell metastasis, cell cycle, cell progression, EMT, autophagy and genome-wide hypomethyltion, mainly via the following mechanisms: (i) SNHG6 functions as miRNA sponges to antagonize the connections between multiple tumor suppressor miRNAs and their target mRNAs. (ii) SNHG6 acts as a scaffold RNA molecule by interacting with EZH2, a subunit of the polycomb repressive complex to influence the expression of downstream effectors of EZH2. (iii) SNHG6 activates JNK pathway, PI3K/AKT/mTOR and TGFβ/SMAD pathway

In a variety of cancers, SNHG6 is highly expressed in cancer tissues in contrast to noncancerous tissues. SNHG6 is also upregulated in the investigated cancer cell lines. Overexpressed SNHG6 arrests cell cycle and reduces apoptosis but promotes migration, invasion, metastasis, EMT, and chemoresistance. The relevant clinicopathological features and underlying molecular mechanisms of SNHG6 in various cancers are summarized in Tables 1 and Fig. 1. The molecular mechanisms can be divided into three categories, as following: (i) SNHG6 functions as miRNA sponges to antagonize the connections between multiple tumor suppressor miRNAs and their target mRNAs. (ii) SNHG6 acts as a scaffold RNA molecule by interacting with EZH2, a subunit of the polycomb repressive complex to influence the expression of downstream effectors of EZH2. (iii) SNHG6 activates JNK pathway, PI3K/AKT/mTOR and TGFβ/SMAD pathway. These studies have focused only on tumor cell proliferation, apoptosis, invasion, and migration of these classic phenotypes, especially cell proliferation. Further studies are needed to explore the phenotypes of SNHG6 in tumors, such as tumor metabolism and immunity escape.

**Table 1:** SNHG6 is an oncogene in tumorigenesis and tumor progression

Cancer type	Function	Cell lines	Assay methods	Isoforms	Molecular mechanism	Reference	Journal
Gastric cancer	Oncogene	MGC-803,AGS,SGC-7901,BGC-823	MTT, colony formation, Cell apoptosis and cell cycle analysis, Migration assay	NA	Downregulating the expression of P27 by recruiting EZH2,and function as a competing endogenous RNA for miR-101-3p	Yan et al. [29]	Cell Physiol Biochem
Breast cancer	Oncogene	BT-474,MDA-MB-231,IR-75-30,T-47-D,MDA-MB-468	CCK8,EdU,Transwell,Tumorigenicity in nude mice	NA	Functioning as a competing endogenous RNA for miR-26a-5p	lv et al. [39]	Biomed Pharmacother
Glioma	Oncogene	T98G,U87,U251,LN-229	CCK8,Cell apoptosis analysis, Invasion assay, Transplanted tumor in nude mice	NA	Functioning as a competing endogenous RNA for miR-101-3p	Meng et al. [43]	Int J Biol Markers
Osteocarcinoma	Oncogene	KHOS,MG-83,U2OS	MTT, EdU, Colony formation, Cell apoptosis and cell cycle analysis	NA	Downregulating the expression of P21 and KLF2	Ruan et al. [45]	Arch Biochem Biophys
Osteocarcinoma	Oncogene	SOSP-9607, MG63	MTT, colony formation, wound healing and transwell assay, cell cycle and apoptosis ananlysis	NA	Functioning as a competing endogenous RNA for miR-26a-5p	Zhu et al. [49]	Cancer Cell Int
Colorectal cancer	Oncogene	Lovo,RKO,Ls174t,DLD1,HCT116	CCK8, Colony formation, Wound healing, Transwell assay, Xenograft model and liver metastasis model	NA	Functioning as a competing endogenous RNA for miR-760	Zhu et al. [23]	Onco Targets Ther
Gastric cancer	Oncogene	MGC-803,AGS,SGC-7901,BGC-823,MKN45	CCK8, Colony formation, Xenograft model in nude mice	NA	Upregulating P21 by decreasing EZH2 and activating JNK	Li et al. [30]	Life sci
Esophageal squamous cell carcinoma	Oncogene	ECA-109,TE-1	MTT, Colony formation, Cell apoptosis analysis	NA	NA	Fan et al. [31]	Oncol lett
Hepatocellular carcinoma	Oncogene	MHCC-77H,HCC-LM3, Huh7, SMMC-7721, BEL-7402	CCK8, EdU, In vivo tumor growth assay	SNHG6-003	Functioning as a competing endogenous RNA for miR-26a/b	Cao et al. [18]	Oncogene
Lung adenocarcinoma	Oncogene	A549,H1299,HCC827,NCI-H358,NCI-H400,NCI-H1650	MTT, Migration and invasion assay, Wound healing, Xenograft model	NA	Functioning as a competing endogenous RNA for miR-26a-5p	Liang et al. [37]	Biomed Pharmacother
Colorectal cancer	Oncogene	HCT116, SW480, RKO, Caco-2, SW620, HCT8, Lovo, HT-29	CCK8, Colony formation, BrdU, Xenograft tumor formation	NA	Repressing P21 transcription through recruiting EZH2 to the P21 promoter	Li et al. [30]	Cell Physiol Biochem
Colon cancer	Oncogene	HCT-116, Lovo	CCK8, Colony formation, Cell apoptosis analysis	NA	NA	Li et al. [30]	Pathol Res Pract
Bladder cancer	Oncogene	RT-4, EJ, BIU-87	Scratch assay, Migration and invasion assay.	NA	Functioning as a competing endogenous RNA for miR-125b and trigger EMT	Wang et al. [26, 42]	Journal of Cellular Biochemistry
Glioma	Oncogene	U87,SHG44, U251, U373-MG	MTT, Colony formation, Cell apoptosis and cell cycle analysis	NA	Repressing p21	Cai et al. [44]	Biomed Pharmacother
Hepatocellular carcinoma	Oncogene	Huh7,HepG2,Hep3B,MHCC97L,HCCLM9, QGY-7701	CCK8, EdU, Transwell assay, Wound healing, Cell apoptosis analysis, Xenograft model	NA	Functioning as a competing endogenous RNA for miR-101-3p and directly binding UPF1 to activate TGFβ/SMAD pathway	Chang et al. [9]	Cancer Letter
Hepatocellular carcinoma	Oncogene	Huh7, HCC-LM3, SK-Hep-1, Hep3B	Ultra-performance liquid chromatography	NA	Functioning as a competitive endogenous RNA for miR-1297 and thereby simultaneously activating 2 positive feedback loops to upregulate MAT2A expression and suppress MAT1A expression	Guo et al. [20]	Cancer Res
Colorectal cancer	Oncogene	HT29, CaCO2, SW480, SW620, RKO, HCT116 and LoVo	CCK8, Transwell assay, Wound healing, Cell apoptosis analysis, In vivo experiments	NA	Activating the TGF-β/Smad pathway via binding UPF1,and functioning as a competing endogenous RNA for miR-101-3p	Wang et al. [10]	Int. J. Med. Sci.
Colorectal cancer	Oncogene	HCT-116, HCT-8, SW-480, SW-620, DLD-1, and HT-29	CCK8, Colony formation, EdU, Transwell assay, Wound healing, Cell apoptosis and Cell cycle analysis, Xenograft model	NA	Functioning as a competing endogenous RNA for miR-214-3p, miR-26a-5p, or miR-26b-5p	Xu et al. [24]	Journal of Hematology & Oncology
Colorectal cancer	Oncogene	HT29, CaCO2, SW480, SW620, HCT116	MTT, Colony formation, Transwell assay, Wound healing, Cell apoptosis and Cell cycle, Xenograft model	NA	Functioning as a competing endogenous RNA for miR-181a-5p	Yu et al. [25]	Cancer Management and Research
Colorectal cancer	Oncogene	HT29, RKO, HCT116	CCK8, Cell apoptosis, Xenograft model	NA	Functioning as a competing endogenous RNA for miR-26a-5p	Wang et al. [10]	Cancer Cell Inter
Colorectal cancer	Tumor suppressor	NCM460, SW480, HCT116	MTT, Transwell assay, Wound healing, Cell apoptosis.	NA	Targeting ETS1 via activating PI3K/AKT/mTOR pathway	Meng et al. [43]	Mol Med Rep
Colorectal cancer	Oncogene	HT29, SW620, HIECs	MTT, Colony formation, Transwell assay.	NA	Functioning as a competing endogenous RNA for miR-101-3p	Shao et al. [12]	BMC gastroenterology
Hepatocellular carcinoma	Oncogene	HL-7702, HepG2, Hep3b, HLE and Huh-7	Cell viability assay, Colony formation, Transwell assay, Cell cycle, Xenograft model	NA	Functioning as a competing endogenous RNA for miR-139-5p	Wu et al. [17]	Cell cycle
Hepatocellular carcinoma	Oncogene	MHCC-97H and HCC-LM3	CCK8 assay.	NA	Functioning as a competing endogenous RNA for let-7c-5p	Chen et al. [19]	Biochem Biophys Res Commun
Esophageal squamous cell carcinoma	Oncogene	EC109, EC9706, KYSE30, and KYSE150	CCK8, Colony formation, Transwell assay.	NA	Functioning as a competing endogenous RNA for miR-186-5p	Du et al. [33]	Dig Dis Sci
Esophageal squamous cell carcinoma	Oncogene	EC9706, EC109, EC1, HET-1A	Cell proliferation, migration and invasion assays	NA	NA	Zhang et al. [16]	Am J Transl Res
Breast cancer	Oncogene	EFM192A, AU565, UACC893, MDA-MB- 415, HS742 T, MDA-MB-231, MCF-7	CCK-8, colon formation, EdU assays, Wound healing, Transwell assay, Cell cycle.	NA	Functioning as a competing endogenous RNA for miR-26a	Li et al. [40]	Pathol Res Pract
Non-Small Cell Lung Cancer	Oncogene	A549, H226, H292, ANP973 and H1299	CCK8, Cell apoptosis and Cell cycle, Wound healing, Transwell assay.	NA	Functioning as a competing endogenous RNA for miR-944 and miR-181d-5p	Geng et al. [34]	Onco targets ther
Non-Small Cell Lung Cancer	Oncogene	A549, H460, and H1299	MTT, Cell apoptosis, Xenograft model.	NA	Functioning as a competing endogenous RNA for miR-490-3p	Dong et al. [35]	Cancer biother radiopharm
Non-Small Cell Lung Cancer	Oncogene	A549, NCI-H23, NCI-H1993, NCI-H522 and NCI-H460	MTT, Transwell assay,Xenograft model	NA	Functioning as a competing endogenous RNA for miR-101-3p	Li et al. [36]	Thorac cancer

*NA* Undetermined

This review focuses on the role of SNHG6 as ceRNA and provide details about SNHG6, its sponging miRNAs, and target genes, which is summarized in Table 2. Given its contributions to cancer development, using SNHG6 and its targets has potential for diagnosis and treatment of cancer. In clinical practice, overexpressed SNHG6 could be a vital biomarker for diagnosis and prognosis of tumor patients. Nevertheless, the chemical stability of SNHG6 in biological samples (e.g., serum) remains unclear. Although extensive researches have been carried out on the contribution of SNHG6 to cancer biology, diagnosis, and prognosis, further studies are needed to shed light on its possible therapeutic intervention.

**Table 2:** Details about SNHG6 functioning as ceRNA in tumorigenesis and tumor progression

Cancer type	miRNA	3′–5′ sequence	PBS to SNHG6	Experimental method	Target protein	References
Gastric cancer	miR-101-3p	AAGTCAATAGTGTCATGACAT	12	Luciferase activity assay, RIP, RNA pull down assay	ZEB1	Yan et al. [29]
Breast cancer	miR-26a-5p	TCGGATAGGACCTAATGAACTT	8	Luciferase activity assay	MAPK6	lv et al. [39]
Glioma	miR-101-3p	AAGTCAATAGTGTCATGACAT	12	Luciferase activity assay	NA	Meng et al. [43]
Colorectal cancer	miR-760	AGGGGUGUCUGGGUCUCGGC	7	Dual luciferase reporter assay	FOXC1	Zhu et al. [23]
Hepatocellular carcinoma	miR-26a	CGGATAGGACCTAATGAACTT	8	RIP, luciferase reporter assays.	TAK1	Cao et al. [18]
Hepatocellular carcinoma	miR-26b	TGGATAGGACTTAATGAACTT	8	RIP, luciferase reporter assays.	TAK1	Cao et al. [18]
Lung adenocarcinoma	miR-26a-5p	TCGGATAGGACCTAATGAACTT	8	Luciferase reporter and RNA pull-down assays	E2F7	Liang et al. [37]
Bladder cancer	miR-125b	UCACAAGUCAGGCUCUUGGGAC	8	Luciferase reporter gene assay	Snail1/2 and NUAK1	Wang et al. [42]
Hepatocellular carcinoma	miR-101-3p	AAGTCAATAGTGTCATGACAT	9	Luciferase assay	ZEB1	Chang et al. [9]
Hepatocellular carcinoma	miR-1297	AAGTTCATCATTCCCT	11	Luciferase assay	MAT2A and FUS	Guo et al. [20]
Colorectal cancer	miR-101-3p	NA	NA	qRT-PCR	ZEB1	Wang et al. [26, 42]
Colorectal cancer	miR-214-3p	UGACGGACAGACACGGACGACA	14	Luciferase reporter assay, RNA pull-down assay, ChIP, RIP	EZH2	Xu et al. [24]
Colorectal cancer	miR-26a-5p	CGGATAGGACCTAATGAACTT	8	Luciferase reporter assay, RNA pull-down assay, ChIP, RIP	EZH2	Xu et al. [24]
Colorectal cancer	miR-26b-5p	TGGATAGGACTTAATGAACTT	8	Luciferase reporter assay, RNA pull-down assay, ChIP, RIP	EZH2	Xu et al. [24]
Colorectal cancer	miR-181a-5p	TGAGTGGCTGTCGCAACTTACAA	7	Luciferase reporter assay	E2F5	Yu et al. [25]
Osteocarcinoma	miR-26a-5p	TCGGATAGGACCTAATGAACTT	8	Luciferase reporter assay	ULK1	Zhu et al. [49]
Colorectal cancer	miR-101-3p	AAGTCAATAGTGTCATGACAT	13	Luciferase reporter assay	β-catenin	Shao et al. [12]
Colorectal cancer	miR-26a-5p	CGGATAGGACCTAATGAACTT	8	Dual‑luciferase reporter assay	ULK1	Wang et al. [10]
Hepatocellular carcinoma	miR-139-5p	UGACCUCUGUGCACGUGACAUCU	7	Dual‑luciferase reporter assay,RIP	SERPINH1	Wu et al. [17]
Hepatocellular carcinoma	let-7c-5p	ATGGAG	6	Luciferase reporter assay	c-Myc	Chen et al. [19]
Esophageal squamous cell carcinoma	miR‑186‑5p	UCGGGUUUUCCUCU–UAAGAAAC	13	Luciferase reporter assay, RNA Pull‑Down Assay	HIF1α	Du et al. [33]
Breast cancer	miR-26a-5p	GAUUACUUGAACGAGGCCAC	8	Luciferase assay, RNA Pull‑Down Assay,RIP	VASP	Li et al. [27]
Non-Small Cell Lung Cancer	miR-181d-5p	UGGGUGGCUGUUGUUACUUACAA	10	Dual-Luciferase Reporter Assay, RIP	ETS1	Geng et al. [34]
Non-Small Cell Lung Cancer	miR-944	GAGUAGGCUACAUGUUAUUAAA	13	Dual-Luciferase Reporter Assay, RIP	ETS1	Geng et al. [34]
Non-Small Cell Lung Cancer	miR-490-3p	GUCGUACCUCAGGAGGUCCAAC	7	Luciferase reporter assay	RSF1	Dong et al. [35]
Non-Small Cell Lung Cancer	miR-101-3p	AAGTCAATAGTGTCATGACAT	13	Dual-luciferase reporter assay,RIP	CDYL	Li et al. [36]

*PBS* predicted binding sites. *NA* Undetermined, *ChIP* Chromatin Immunoprecipitation, *RIP* RNA Immunoprecipitation

## Data Availability

Data sharing not applicable to this article as no datasets were generated or analysed during the current study.
